# "The Hospital" Nursing Mirror

**Published:** 1901-01-26

**Authors:** 


"The Hospital" Nursing Mirror.

Being the Nursing Section of "The Hospital."

[Contributions for this Section of "The Hospital" should be addressed to the Editor, "The Hospital" Nursing Mirror, 28 & 29 Southampton Street Strand, London, W.C.]

### Queen Victoria and the Nursing Vocation.

No section of the community will feel the death of the beloved Queen more deeply than those whose vocation is the ministry of suffering. We do not doubt that nurses throughout the country, and beyond the seas, followed with keen anxiety and tender attention the course of the illness which, in spite of every effort of human skill, terminated fatally on Tuesday afternoon. They were better able than the vast majority of people to stand in imagination by the sick-bed of the illustrious lady, and to enter into the feelings of hope and despair which must at times have alternated in the hearts of those who watched by her side. While the undivided nation mourns for the Queen-Mother, and the civilised world does homage to her goodness and greatness, nurses have special cause to offer a tribute of their own to her memory, and to lament the fact that she has passed away. Throughout her life she manifested the warmest sympathy in the movement for the elevation of nursing, and was proud to count Florence Nightingale amongst her personal friends. It was a real pleasure to her to be able to lay the foundation-stone of St. Thomas's Hospital, where the Nightingale School commenced its career. Under her auspices in 1863 the Order of the Royal Red Cross was instituted, and one of her last acts before her illness was to award the decoration to a nurse who earned the distinction by her services during the siege of Tien-tsin. Only a few weeks ago, when the Queen was at Windsor, she found time, in spite of her numerous engagements, to receive two nursing sisters who tended the sick and wounded in the yet more memorable siege of Mafeking. No phase of the war in South Africa escaped her attention, and her sympathy for the nurses engaged in succouring the troops was frequently and freely expressed. There is good reason to believe that she intended to recognise their work by the gracious bestowal of some token of her approval. It was always a source of gratification to her to confer a mark of Royal favour upon a nurse, whether it had been earned by devotion among the wounded in Egypt, the fever-stricken peasants of Inneskea, or in the plague hospitals of India. As recently as January 1899 she conferred the distinction of Serving Sisters of the Order of the Hospital of St. John of Jerusalem in England upon nine plague nurses; and in the same year she generously responded to an appeal of the Up-Country Nursing Association, of which she was Patron, for funds to enable the committee to send out eight more nurses to labour among the Europeans in her Indian Empire. To Queen Victoria's liberality the existence of one of the most useful nursing institutions in the kingdom is due. At her desire the surplus of the Women's Jubilee Offering in 1887—a sum of no less than £70,000—was allocated to the beneficent purpose of founding an organisation for promoting the nursing of the sick poor in their own homes. The Queen Victoria Jubilee Institute for Nurses will remain an enduring monument of the practical interest which Her Majesty manifested in the progress of nursing; and the rapid development of the district nurse in all parts of the kingdom is one of its notable outcomes. But "Queen's nurses" themselves have increased and multiplied. There is a branch of the Institute in Scotland, and in Ireland; and when the Queen visited the sister island last year, she excited unbounded joy among her Jubilee nurses by receiving them as well as the matrons of the Dublin hospitals. None of the 398 Queen's nurses who had the privilege of being entertained by her at Windsor on July 11th, 1896, will have forgotten either the function, or the characteristic welcome of their Royal hostess. But her words on that memorable day will be eagerly read by thousands of nurses at the present moment. "I am very pleased," she said, "to see my nurses here to-day, and to hear of the good work which they are doing, and which I am sure they will continue to do." Her own enthusiasm never slackened. The additions which were annually made to their number always underwent her careful scrutiny, and no name appeared on the list without her direct sanction. She honoured the calling, and did her best to guard it from dishonour. Now and again she sent, through the medium of the Jubilee nurses, messages to patients whose cases had attracted her attention; and in December 1897 the heart of a poor old woman at Tunbridge Wells was made glad by an intimation of her Sovereign's satisfaction at the manner in which she had been tended. Thus, in a variety of ways, she sought to dignify and ennoble the work of nursing; and her efforts will not die with her. They will live on, bearing fresh fruit in many lands and countless homes, a rich heritage for all who can be stimulated and inspired by high example and resolute endeavour.

### Notes on News from the Nursing World.

### THE PRINCESS OF WALES AND HER HOSPITAL SHIP.

With her customary thoughtfulness and unfailing kindness of heart, the Princess of Wales—now the Queen-Consort—has sent by Sister Chadwick of the Army Nursing Service, a number of small packages containing articles of clothing and sundry comforts for the patients in some of the hospitals in South Africa which Miss Chadwick will visit. In recognition of the work of Miss Chadwick, who was in charge of the Princess of Wales's hospital ship during the whole period of the vessel's service between England and South Africa, Her Royal Highness has presented her with a very handsome gold and enamel cross. The Princess of Wales has also presented each patient at the Gables private military hospital with a pocket-case and a cartridge pencil bearing the engagement at which the recipient was wounded. The Princess's hospital ship has now made its last voyage to the Cape, and the hospital which Sir Alfred Cooper has equipped and maintained is to be closed shortly. Sir Alfred has received a silver cigarette case from soldier patients and Lady Cooper a silver card case, while the nursing staff also received presents.

### NURSES FROM THE FRONT.

The s.s. *Dunera*, which arrived last week, had on board Sister Potts (A.N.S.); Sisters Gibb, Snell, Sands, Moxon, Willett, and Roberts, of the Army Nursing Service Reserve; the last is marked on the official roll as a volunteer. Sisters Sands, Tripp, Templeton, Asmans, Grant More, and James (volunteer) were invalided home. The s.s. *Formosa* brought Sisters Florence Baker, Helen Thomson, and Ada Murton (A.N.S.R.), and Sisters Eliza Bole and Mary M. Knox.

### SCOTTISH RED CROSS HOSPITAL NURSES.

Last week a number of the nurses of the Scottish Red Cross Hospital were welcomed home by a representative gathering at the Windsor Hotel, Glasgow, the others having again been called up for service. One of the speakers on the occasion was Sir David Richmond, a member of the Hospitals Commission, who expressed his delight at seeing some of the nursing sisters whose acquaintance he had made in South Africa. He said that, "a more noble work he never saw any one engaged in." Dr. Clark also bore testimony to the value of the nurses' labours, and rejoiced that they had been so much appreciated that several of the sisters had been sent out again. The proceedings throughout were of a most enthusiastic character.

### LEEDS NURSES AND THE WAR.

Application has been made to the Leeds General Infirmary for eight nurses for the seat of war, but it is not thought probable that the committee will be able to spare them. Several old Leeds Infirmary nurses are now at the front. One of them had a curious experience recently. She found it necessary to send for the medical officer in order to certifiy as to the quality or condition of some milk. She had never seen him, and consequently when his voice was heard at the door ordering in stentorian tones that the milk should be brought to him for his inspection, she owned to feeling a trifle nervous. On turning round to have a look at him, however, great was her astonishment to find that the terrible medical officer was a doctor with whom she had long worked at the Leeds Infirmary. The meeting under such strange circumstances so far away from home was, as may be supposed, mutually pleasurable. Miss Wilcock, who has just left England for South Africa, was trained at the Leeds Infirmary.

### ROBBING AN ARMY NURSE OF HER LUGGAGE.

That a soldier who was engaged in the siege of Mafeking should have disgraced himself by robbing an Army nurse who nursed wounded soldiers at Winburg is very sad. This, however, was the substance of the story told in the Southwark police court the other day by Miss Mary Rogers. Like herself, Morgan, the soldier, had been invalided home, and he returned in the same vessel as she did to England. They travelled from Southampton in the same train, and at Weybridge Miss Rogers asked Morgan to look after her luggage at Waterloo, whence it had been sent on by accident, and forward it on to her address. He promised to do so, but took it with him to Lancashire instead. It was subsequently traced by the police, and Morgan admitted in court that he had stolen it. The heartlessness of the theft was all the greater because Morgan knew that Miss Rogers was in delicate health, and had no home, as well as that her brother was at the front. She naturally supposed that a soldier of the Queen, who had fought bravely for his country, was incapable of robbing an Army nurse.

### IN MEMORY OF A HEROIC NURSE.

At Longmoor Lane Cemetery, Liverpool, an impressive ceremony took place the other day, the occasion being the unveiling of a handsome memorial stone at the head of the grave of Nurse Christina Jackson. The inscription tells its own tale, and is as follows:—"In loving memory of Christina Jackson, who died April 12th, 1900, aged 34 years; daughter of Donald and Sarah Jackson. This stone is erected to her memory by her shipmates in the victualling department of the s.s. *New England* to mark her heroism in nursing during a severe sickness on board, from which sickness she afterwards died. Let her dear memory serve to make our faith in goodness strong." At the time of her premature death Miss Jackson was one of the Queen's Jubilee nurses.

### CRISIS AT PORTSMOUTH INFIRMARY.

The matron and assistant matron of Portsmouth Workhouse Infirmary have resigned, the former on the ground that her authority was undermined, and her colleague because she was in sympathy with the matron. The resignations have been accepted by the Portsmouth Board of Guardians, who have also determined to give the retiring matron and assistant matron the best of testimonials. This, however, increases perplexity as to the real cause of the crisis, and we understand that in the town of Portsmouth there is a strong feeling that some further explanation than has yet been made is due to the public. In this instance there was no question of an untrained matron, both Miss Court, the matron, and Miss Johnson, the assistant matron, being thoroughly qualified nurses.

### "WHY WILL NURSES BE SO FOOLISH?"

A private nurse relates, in our correspondence column, an incident which merits attention. Her present patient tells her that a short time ago he was staying at a leading London hotel and sent for a nurse. Almost the first thing she said on her arrival was, he states, "Where am I to have my meals?" His rejoinder, after a little consideration, was, "With the housekeeper, I should think." Whereupon, he adds, the nurse immediately left him. Our correspondent justly inquires, "Why will nurses be so foolish?" In fact, such conduct is worse than folly. The nurse whose first thought is about her meals is not very well suited for her profession. It is, of course, essential that she should have good meals at regular hours, and if she does not get them, she has a right to complain. In this instance we have no doubt, as our correspondent suggests, that the nurse whould have had her meals sent to her own room. If her patient lacked tact in his proposal about the housekeeper, she displayed temper in resenting it as she did—the kind of temper which no nurse should permit herself to exhibit. As to the idea of nurses in uniform appearing at *table d'hôte*, hotel managers are obliged to consider their customers; and there cannot, in a well-ordered establishment, be the slightest difficulty in taking the meals of a nurse to her own room.

### SISTER BOYD-CARPENTER'S WEDDING.

Our readers will be interested to know something about the career of Mrs. W. J. E. Davies (Miss Jessie Peers Boyd-Carpenter), and the matron of St. Thomas's Hospital has been kind enough to supply the following details:—Miss Boyd-Carpenter entered St. Thomas's Hospital as a probationer in October, 1892. In 1895 she was appointed sister of the "Christian" female medical ward, and in January, 1898, sister in charge of St. Thomas's Home, which position she occupied until June, 1899, when she left St. Thomas's. The matron spoke of her as a "nice bright sister," very fond of her work, and added that the wedding at St. George's, Bloomsbury, was a very pretty one. The bride looked very nice in a dress of ivory satin, with sleeves of embroidered chiffon, and a long Court train of white satin falling from one shoulder and draped with festoons of white chiffon and orange blossoms. Her veil was of Brussels lace, and her only ornament a pearl and diamond brooch. Dr. and Mrs. Davies are spending the honeymoon at Brighton.

### THE NEW MATRON OF MELBOURNE HOSPITAL.

Miss Amy L. Burleigh, the new matron of Melbourne Hospital, after her training at St. Bartholomew's Hospital, was first night sister at the City of London Hospital for Diseases of the Chest, and then home sister. During the absence of the matron, Miss Beatrice Jones, in South Africa, Miss Burleigh acted as matron; and on the return of Miss Jones, at the expiration of eight months, resumed her duties as home sister.

### A BRISTOL SCANDAL.

With reference to our comments, in a recent issue, on the withdrawal of a nurse from the poor and populous district of Bedminster in Bristol we are informed "that the late nurse had foreseen what would happen, and had trained one of the parishioners to enable her to undertake part of the work she herself was leaving." But this is an extremely unsatisfactory state of affairs. No parishioner of Bedminster who has not received the proper training of a nurse is competent to discharge the duties of one; and the temporary arrangement which has been made accentuates, rather than otherwise, the scandal that the citizens of Bristol should be so wanting in public spirit as not to provide the funds for the support of a qualified nurse in a part of the city where she is particularly needed.

### A SUGGESTION FOR THE BUXTON NURSING ASSOCIATION.

An adverse balance, however small it may be, is always a matter for regret; but the fact that there was £6 10s. 6d. on the wrong side at the end of the year need not vastly trouble the Committee of the Buxton District Nursing Association so long as they continue to work in harmony and the services of their nurses are so warmly appreciated. In fact, at the annual meeting the other day the Chairman, Dr. Turner, alluding to a proposal that the Board of Guardians might contribute to the funds, said there was no longer any necessity for anything of the kind, and that the people of Buxton were both willing and anxious to render what assistance the poor of the district required. In these circumstances we hope that the suggestion of Dr. Braithwaite may speedily be adopted. Dr. Braithwaite, who lived in Yorkshire before he went to Derbyshire, mentioned that at Richmond in the former county they had one or two beds in the nurses' home to which serious cases could be sent, and which did not materially interfere with the outside work of the nurses. He expressed a wish to see something of the same sort carried out in Buxton, and the Chairman and other speakers promptly acknowledged the value of the suggestion, one of them adding that if it were acted upon the subscriptions would probably be increased. We have not the least doubt that they would. The liberality of the public is invariably quickened by the display of enterprise in a good cause.

### DISTRICT PATIENTS' TREAT.

A number of children were entertained on Saturday afternoon at Carnforth Lodge by Miss Curtis and the nurses of the Hammersmith and Fulham District Nursing Association. Invitations had been given to children who were patients and to those who had "been good to their mothers" during illness. Two little boys were brought lying down; one, a bright little fellow of about seven, who had been eight months in a spinal box. "There goes the wounded soldier!" the other boys said, as he was carried into the room where the tree was waiting to have its fruit plucked. It took the nurses quite an hour to distribute the little gifts, sweets and crackers, and at six o'clock, laden with many packages, the guests departed to their homes. "He has been looking forward to it all day," said one of the mothers; "he was so afraid we should be late."

### PROGRESS AT BARRY.

The annual meeting of the Barry District Nursing Association was held last week. A very satisfactory report was presented, showing that during the year 473 cases were treated in the Home belonging to the Association, while the total number of visits paid by the staff was 11,597. Until last March the Association and an accident hospital of seven beds were under the same committee and the same matron. But the accident hospital has since been taken over by the Town Council, and is now rate supported. The Association remains a voluntary institution, largely supported by the railway men and employés in the different docks. The large and pleasantly situated Home was built in commemoration of the Queen's Diamond Jubilee. Each nurse has a separate bedroom; there is a good-sized general sitting-room, and a very large dining-hall, which is also used as a committee-room. The superintendent has a roomy office and a comfortable sitting-room. The Railway Company grant free passes on their line to all the staff, which is a great privilege and a help, both for work and pleasure. The income from subscriptions, workmen's pennies, &c., is sufficient for the yearly expenditure, but there is a heavy debt on the building and furnishing, which it is greatly desired to liquidate. Miss Sykes, formerly superintendent of the combined institutions, is now matron of the accident hospital, and Miss Christine Aldis has been appointed superintendent of the District Nursing Association. Miss Aldis has enjoyed varied experience, and has taken up her work with much earnestness and hope.

### A NOVEL PROPOSAL.

At the annual meeting of the Strabane District Nursing Society the Duchess of Abercorn said she had often wondered, thinking of the great work done by the organisation, how the people of Strabane did before it was started. To say that her Grace's wonder is often shared by other people connected with other societies is only another way of stating that the district nurse is rapidly becoming indispensable. An interesting proposal was made at the Strabane meeting, that a committee of ladies affiliated to the Nursing Society should offer prizes for the best kept houses of the working classes, and it was urged that this would be "of the greatest assistance in the furtherance of the work of the nurse." We are not at all sure that it would. While it is of the utmost importance that the dwellings of the working classes should be as clean and as comfortable as possible, it would not be sound policy to take any course which would cause the patients and their friends to regard the nurse in the light of an inspector. If the prize system is carried out, it should be clearly understood that the nurse at Strabane has no kind of responsibility for the awards, and that her obligations begin and end with nursing.

### A FALSE REPORT.

"There was no fire at Charing Cross Hospital," said Miss Gordon to our representative, who called to enquire into the cause of the "outbreak" referred to in one or two of the evening papers. "Mr. Reade, the Secretary, has written to contradict the report. All that happened was that one of the workmen in the engineers' new workshop set fire to a piece of canvas, and it was out so quickly that we did not even need our own hose. Someone, however, gave the alarm of fire; several engines dashed up and a crowd collected. There was naturally 'no panic'; but the inmates of the hospital were much interested in the arrival of the engines. It is rather tiresome, and I think the reporters must have been hard up for news. Several such reports would give a hospital a bad name, for the public would think that fires were always taking place."

### QUEEN'S NURSES AT DARLINGTON.

The ninth annual meeting of the Darlington Queen's Nurses' Association was held last week in the Council Chamber, Darlington, the Mayor presiding. According to the report of the Committee a very high average number of visits was paid, no fewer than 283 per week, or an aggregate of nearly 15,000 during the past year. This is the most convincing proof that could be given of the value attached to their services by medical men and patients alike. The financial condition of the Association during the past year was a subject of much anxiety. A special appeal was issued on November 21st and resulted in £100 being collected. There had been two visits from the Inspector sent by the Queen Victoria Jubilee Institute, whose reports were very satisfactory. The Chairman and other speakers bore testimony to the good work which had been done by the staff of four nurses. The home started work on April 1st, 1892, at the suggestion of the late Mr. A. Pease, M.P., with one trained nurse. The staff now consists of four, the annual cost of the home being a little over £400. The founder contributed £100 per annum towards its funds, and his widow, whose interest in the Association is great, is President of the Committee.

### A CRY FROM FAR ACHILL.

It is not, we learn from the Secretary of Queen Victoria's Jubilee Institute for Nurses, because the Congested Districts Board are in "a sad state of penury" that they have ceased to maintain the nurse. The Board's own funds, our correspondent says, cannot and have not been applied for the purpose; but "the maintenance of the nurse was supplied from a special fund at the disposal of the Board, and this fund was exhausted in March last." Our correspondent continues: "It is entirely owing to the Congested Districts Board that the nurse is in Achill at all; they initiated the work, and they have now offered to provide a cottage for the nurse, and are at present making an allowance towards her lodging expenses." No reproach can, in these circumstances, attach to the Board. And as it seems quite impossible that the money required can be raised locally, those who wish to help in the good work of securing the stay of the nurse in Achill Island cannot do better than send in their contributions to the Secretary of the Queen Victoria's Jubilee Institute for Nurses, St. Katharine's Precincts, Regent's Park, N.W.

### LEGAL PROCEEDINGS AT ARMAGH.

The guardians of the Armagh Union are not to be allowed to defy the Irish Local Government Board with impunity. Proceedings are to be taken to test the legality of the employment of a person as nurse at the infirmary who is considered by the Local Government Board to be insufficiently trained, and whose sanction to the appointment has, for that reason, been withheld. This is a point of the utmost importance, and if the result of the trial should be to prove that the Irish Local Government Board do not possess the power they claim to exercise, they will certainly have to be invested with it.

### SHORT ITEMS.

Sister E. C. Laurence, who only arrived from South Africa in the *Canada* a fortnight ago, has left again in the same vessel, but at the time of starting was not aware of her destination.—Another engagement of an army nurse is announced. Miss Florence Learmouth, who went out to South Africa at an early stage of the war, will shortly be married to Major Kemp.—Miss Lucy Yates has just passed through the press her small book, *Convalescents' Diet*; it is written from the practical point of view of a nurse and cook, and will be published by H. Virtue and Company, Limited.—Miss Agnes M. Canner, of Hackney Union Infirmary, who has just completed her three years' training, midwifery included, has obtained the L.O.S. certificate. The ball to nurses of the Edinburgh Fever Hospital, which was fixed to take place this week, has been postponed in consequence of the Queen's death.—Miss F. E. Scott, who has just gone to South Africa as a member of the Army Nursing Service Reserve, is not, were are informed, on the staff of the Nurses' Co-operation.

### Lectures to Midwifery Nurses.

By ROBERT JARDINE, M.D., &c., Physician to the Glasgow Maternity Hospital. Examiner in Midwifery to the University of Glasgow, &c.

(*Continued from page* 196.)

### SOME OF THE BLADDER COMPLICATIONS MET WITH DURING PREGNANCY.

The bladder, as you know, is the reservoir in which the urine is stored. The kidneys are the organs which excrete the urine, and from each kidney a tube, the ureter, carries the urine into the bladder where it is retained for some hours. As soon as the bladder becomes full, under ordinary conditions the woman feels a desire to empty it, and she can do this by an effort of will. The urine is voided through the urethra. If she is unable to attend to the call of nature at once she can resist the desire even for some hours, but she will suffer considerable discomfort. As a rule women can retain their urine much longer than men.

The bladder, as you know, lies in front of the uterus. The normal position of the fundus of the uterus is forwards, and as soon as the organ begins to enlarge in pregnancy it comes to press upon the back wall of the bladder. Frequency of micturition in the early months of pregnancy is very common. In fact, it may continue throughout the whole pregnancy; but it is commoner at the beginning and towards the end of pregnancy than during the middle months.

*Retention of Urine caused by a Posterior Displacement of the Uterus.*—I wish to impress upon your minds that if a woman complains of constant dribbling of urine when she is about three months pregnant, she will in all probability not be suffering from incontinence, but from the very opposite condition, viz. retention of urine. You must not be misled by her statement that she is passing plenty of urine, because she is almost sure to tell you this. You should palpate the abdomen. A large rounded tumour, much the shape and feel of a pregnant uterus will be felt. The top of it may be as high, or higher, than the umbilicus. If she is only three months pregnant this cannot be the uterus. It will be an over-distended bladder. On making a vaginal examination a rounded, somewhat softish body will be easily felt in the posterior fornix, and the cervix will be high up and to the front behind the symphysis. The rounded swelling is the body of the pregnant uterus, which is turned back into the hollow of the sacrum. The cervix is pushed forwards and presses against the neck of the bladder and urethra, and thus prevents the bladder being emptied. Fortunately it does not absolutely block the passage, so as soon as the bladder becomes overdistended constant dribbling of urine begins. If this did not happen the bladder would burst, and such an accident has occurred, but this is exceedingly rare. This constant dribbling of urine may very easily be mistaken for incontinence, while in reality it is due to retention. It is a serious condition. By the fourth month the fundus of the uterus should rise out of the pelvis, but if it is turned back under the promontory, it cannot do this. In a very few cases it may come right, but such a natural termination cannot be looked for. As the uterus increases in size, the pressure upon the surrounding parts becomes greater and greater. The perineum may be so pressed upon as to cause it to slough and even the wall of the uterus may become gangrenous. In some cases an abortion occurs and the woman gets relief in that way.

*Treatment.*—The first thing to do is to relieve the bladder by drawing off the urine. This should be done with the strictest aseptic precautions. There may be considerable difficulty in passing a catheter and a long flexible one may have to be used. If the bladder is very much over-distended it should not be entirely emptied the first time. If it is completely emptied there may be alarming hæmorrhage from the mucous membrane lining it.

The treatment of the displacement of the uterus is not for a nurse to attempt, but she should know about it. The bowels should be thoroughly cleared and the patient left to rest until the following day, the catheter being passed when necessary. If the uterus is incarcerated or jammed below the promontory chloroform will be necessary. The bladder should be completely emptied. In a case which is not fixed replacement may be done by placing the patient in the knee-elbow position. The vagina is then opened and, as the air distends it, the uterus may fall forwards into its right position. If it does not do so, two fingers should be passed into the rectum and firm pressure be made on the fundus, taking care to push it up one or other side of the promontory. This manipulation may be assisted by grasping the cervix with forceps and pulling it down. In some cases it may be replaced with the patient in the lithotomy position by pushing up the fundus by the side of the promontory by means of the middle finger in the rectum and the forefinger in the vagina. Traction on the cervix will also help here.

If it is so firmly fixed that pressure from below fails, it may be relieved by opening the abdomen and pulling it up. This operation has now been done several times with complete success, and the patients have gone on to full time. An abortion may be induced. The cervix is so high up that there will be difficulty in getting a sound in. If this cannot be done an aspirator trocar and canula may be pushed through the posterior wall and the liquor amnii drawn off. This will lessen the size of the uterus and bring on an abortion, but it is a somewhat risky proceeding.

When the uterus is replaced a pessary should be introduced and left in for a month or so, until the fundus is well up into the abdominal cavity. The patient should be kept very quiet for a few days, and an opiate should be given immediately after the manipulations to prevent an abortion occurring.

(*To be continued.*)

### Appointments.

ROYAL ISLE OF WIGHT COUNTY HOSPITAL.—Miss Emily E. C. Antram has been appointed matron. She was trained at the Royal County Hospital, Winchester, where she afterwards remained six and a half years.

INTERNATIONAL HOSPITAL, NAPLES.—Miss von Berg has been appointed matron. She was trained at the Seamen's Hospital, Greenwich, and for the past nine months has been ward sister at Kingston Union Infirmary.

PLYMOUTH WORKHOUSE, HOSPITAL.—Miss Mary Holliday has been appointed superintendent of nurses. She was trained at Blackburn Infirmary, and has since been lady superintendent of the Parish Hospital at Paisley. She holds the L.O.S. certificate.

LADYWELL WORKHOUSE INFIRMARY.—Miss Florence Capes has been appointed assistant matron. She was trained at St. George's Infirmary, Fulham Road, London, and has since been charge nurse for six years at South Metropolitan District Schools, and charge nurse at St. Olave's Infirmary three and a half years.

ST. HELENS BOROUGH SANATORIUM.—Miss Burgess has been appointed matron. She was trained at Adelaide Hospital, Dublin, and has since been charge nurse at private hospital, Dublin; night superintendent at Adelaide Hospital; sister in charge of private hospital, Dublin; assistant matron of fever hospital, Dublin; and matron, County Meath Infirmary, Navan, Ireland.

### Nursing at the Temporary International Hospital, Peking.

By THE MATRON.

On June 21st, the day after the siege began, a temporary hospital was started in the British Legation, the "Chancery" building, or Offices of Legation, being given up for the purpose. We began with one ward and two beds in the operating room and received the Interpreter to the German Legation, who was seriously wounded at the time the Minister was killed, as our first patient. But the number of wounded increased quickly, and one office after another was turned to hospital uses.

### The Supplies.

As far as possible the patients were warded according to their nationality, which made interpretation work easier, and one ward was reserved for officers and civilians. Lady Macdonald supplied a good deal of the furniture, and many sent mattresses, pillows, and linen, and some who had small iron and camp bedsteads gave them up and slept on the floor themselves—large bedsteads were also offered but found impracticable on account of room; box and spring beds, however, were gratefully accepted, as they raised the mattresses from the floor, but a good proportion of them had nothing underneath. For under-pillows we collected the straw envelopes from wine bottles, cut the threads at the pointed end, and laid them two deep in a case. The linen lent was not nearly sufficient, but the whole contents of one of the foreign shops had been brought into the Legation and thence we obtained a supply of towels, yards and yards of calico, &c., which some of the ladies made into sheets, draw-sheets, pillow-slips, shirts, and even some aprons for doctors and nurses. The same store provided the hospital with cups, plates, cutlery, pots and pans of all kinds for ward and kitchen use.

### The Accommodation.

The reading-room made an excellent operating theatre, and after the two bedsteads were removed there was space for two operating tables, and small tables with sets of bowls, dressings, &c., to each one, so that two operations could be carried on at the same time. Two small washstands, cupboards for linen and dressings, and a writing table completed the furniture. The drugs and dressings were mainly supplied by Dr. Poole and Dr. Velde, of the British and German Legations respectively, who were in charge of the hospital.

### The Staff.

The nursing staff consisted of one English and four American lady doctors, who most kindly volunteered their services, the matron, one other trained nurse, several ladies of various nationalities, two gentlemen as honorary stewards, who proved most efficient helpers, and the English and Foreign sick berth stewards. This may seem rather a large staff for an average of forty patients, but the circumstances and strain were exceptional. Four nurses were told off for night work, two on duty every alternate night, and the day nurses came on twice a day for four hours at a time.

### Outline of a Day's Work.

The night nurses had the patients washed by 8 a.m., when the day nurses came on duty, gave breakfasts, took temperatures, made beds, and had the wards tidy by 10 o'clock, ready for the doctors' round. Then came the theatre work. All dressings were done there, except for a few who could not possibly be moved, and we thought ourselves fortunate if we got through by 1.30 p.m.; but it frequently happened that some were postponed till the afternoon, especially at times when the newly wounded came in fast, as on the day when a Russian was brought in who had drunk strychnine by mistake and was lying on the floor, while there was a serious case on each table, and a third wounded on a stretcher outside.

Dinner at noon, tea at 4 p.m., temperatures taken, doctors' round again at five o'clock, supper at 6.30—thus early because all lights had to be extinguished betimes or kept turned low on the floor—medicines and night-draughts given, and the night-nurses came to the relief at ten o'clock. In the theatre the windows were shaded at night by thick straw blinds, as it was necessary to keep a good light there.

### Muslin Curtains for Dressings.

Towards the end we began to be short of dressings, and instead of medicated gauze we used muslin curtains, also muslin bags filled with peat, broken small, and when peat failed had recourse to sawdust. These were all well sterilised, a fresh supply each day, and absorbed fairly well, thus helping the wool to last out. Chinese wool was used for padding splints, and laying outside dressings when hæmorrhage was feared, as we were very short of mackintosh. The wounds healed wonderfully well, and there were no cases of erysipelas. We had only three typhoids, but several were admitted for a few days with fever or diarrhœa.

### The Difficulties.

Perhaps the two chief difficulties were keeping out the flies, and getting the laundry work done. There were three coolies told off for the latter work—of course it was only washing—ironing was a luxury unknown! but the men had no taste for the work, and were not easy to keep at their post. A number of mosquito nets were lent for the beds, but in the theatre the flies were terrible, until it was suggested to take the glass out of the windows and to nail net curtains tightly across instead; also to put the door on swing hinges, and to replace the panels with net. Then the flies were switched out with paper mops, and we were comparatively free.

Worse still than the house fly, was a green one, a little larger, which lays eggs wherever it settles, especially in blankets, and in some few cases got in under the patient's dressings. Creolin was the best antidote for this.

The wards were deprived of some ventilation by the barricades; one window after another had to be banked half way up with sand-bags as the firing became fiercer; some shots and pieces of shell did find their way into the hospital, but no one was injured by them.

### "Pony" Food.

The food department was skilfully managed by three ladies and a Chinese cook, and "pony" became a savoury dish, either as stew, mince, rissoles, curry, &c.; it also made good soup. Sometimes a scraggy sheep was killed, and the hospital had its special share, but "pony" for hungry people was more satisfying.

Eggs were very scarce; of fresh milk there was none, but the condensed milk lasted out to the end, and there was a good supply of canned fruits, some jam, rice instead of vegetables, and white flour used for the bread. Certainly the best of everything was always willingly surrendered for the hospital.

### Ophthalmic Nursing.

By a Sister.

### I.—CARE OF OPERATION CASES.

The most important operations in eye surgery, regarding them from the nursing point of view, are extraction of senile cataract, linear extraction, iridectomy, sclerotomy, paracentesis, enucleation of the globe, and advancement for squint.

There are also a number of minor operations, the nursing of which do not require any special mention here. The day before the operation the patient should be given an aperient, and it is an excellent plan with cases to be operated on, more especially those of senile cataract, to fully explain to the patients how they must behave both during the operation and afterwards. It usually gives the patient great confidence if he is told beforehand that he will have drops put in to prevent him feeling pain—at least, nothing more than a pricking sensation—during the operation. It must also be impressed upon him that after the operation is over and he is in bed a great deal depends upon himself during the first few days whether the operation will be a success or not.

### Preparations.

All drops and dressings must be sterilised before the operation. The steriliser, or its substitute, must be boiling to be in readiness for the instruments; also a tray or dish containing 1 to 40 solution of carbolic acid to place them in. The nurse will have to prepare some very small pointed swabs made of absorbent cotton-wool that has been wrung out of hyd. perchlor. 1 in 5,000, in addition to some larger round ones, and she will have previously seen that everything in the operating-room is scrupulously clean, not forgetting to disinfect her own hands in weak carbolic acid lotion before touching the eye to be operated on. The patients must be prepared in their ordinary bed garments and a warm dressing-gown. Women's hair should be either plaited at each side of the head or twisted on the top, to facilitate the dressing of it without moving the head. In all operations where the eyeball has to be opened the face surrounding the affected eye must be well washed with hot water half an hour before the surgeon is expected. The eye must then be thoroughly bathed with an aseptic lotion.

If the operation is to be done under cocaine, the first drop (4 per cent. solution) should be put in a quarter of an hour before the appointed time for the surgeon's arrival, and repeated four times at intervals of a few minutes, unless the surgeon has left instructions of a different nature.

### Senile Cataract Operation.

After senile cataract extraction the patient lies on his back until given permission to change his position by the surgeon, which is usually in about three days' time. During that period he must not sit up for anything, and must be fed with slop diet, and should be cautioned against coughing or sneezing. If the nurse is directed to put drops or lotion into the eye, it can be done without disturbing the upper lid over the region of the wound, by drawing down the lower lid and dropping the liquid into the inner canthus. The most rigid aseptic precautions must be observed in dressing the patient's eye. Should there be any pain after the first three or four hours have elapsed, it ought at once to be reported to the surgeon.

### If Complications Should Arise.

The nurse must also watch for any rise of temperature, puffiness of the lids, or the presence of discharge, all of which are dangerous symptoms.

Both eyes are always bandaged for the first two or three days after cataract extraction; this sometimes causes delirium, especially if the patient is very old and atropine is being used. On the first appearance of this complication the nurse should at once send for the surgeon's instructions how to act.

He may possibly order the good eye to be unbandaged and a sleeping draught, as any struggle with the patient would be most injurious to the eye. It is to be hoped, however, that none of these complications will occur, and that the case will progress favourably until the fourth or fifth day, when, if the wound be healed, the patient is usually considered convalescent. It is not advisable during the first three days to give an aperient unless ordered, as it is better for the bowels to remain "locked" until the wound has healed

### Cataract in Young People.

In young people the cataract is broken up and dissolved by a series of small operations called discission or needling. These operations are also performed for high myopia, and the nursing is the same in both cases. The patient, if all goes well, is up in a couple of days, and does not require to be kept strictly on his back. The nurse must watch for any complaint of great pain or vomiting, as these are sure signs that the surgeon ought to be sent for. He may possibly relieve the tension of the eye if he finds it is tight or hard by the "linear operation." The nursing after this operation is the same as after cataract extraction; also in iridectomy, sclerotomy, or paracentesis the same rules are observed for at least the first twenty-four hours after the operation.

### Cases of Enucleation and Evisceration.

In enucleation of the eye, which is done under chloroform, there is very seldom any after-trouble beyond occasionally reactionary hæmorrhage, which can generally be checked by fresh dressing and pressure. In cases of enucleation and evisceration the temperature should be recorded both before and after the operation.

In advancement for squint the patient must be kept quiet for three or four days until after the stitches are removed.

### Other Cases.

Tenotomy, for squint, does not require much nursing. The bandages are usually removed in a few hours' time, and the patient allowed to go home the same day or next morning. Operations on the lids or the duct do not, as a rule, want any special after-treatment beyond carrying out the instructions of the surgeon and reporting any increased uneasiness on the part of the patient.

### Follow the Surgeon's Plans.

It must, of course, be understood that before relying on this rough sketch of the nursing of eye operation cases, the nurse must be careful to ascertain that it is the plan of nursing that the special surgeon she is working under approves of. For on the exact period of time the patient is kept in bed, the diet, the different kinds of dressings, and the various methods of application the experienced ophthalmic nurse will find that every surgeon has a different system which she must carry out.

### Presentations.

The R.M.S. "Tokomaru."—Miss I. M. Bethune, who left the Chelsea Hospital for Women a few months ago to proceed to New Zealand, has been the recipient of a handsome present, in the shape of an illuminated address and a purse of sovereigns, from the Captain, officers, engineers, crew and passengers of the R.M.S. *Tokomaru*, for services rendered voluntarily in connection with serious cases of illness and accident on board the steamer during the voyage. The presentation was made by Mr. J. Maxwell, Commander of the R.M.S. *Tokomaru*, off South Australia.

### To Nurses.

**We invite contributions from any of our readers, and shall be glad to pay for "Notes on News from the Nursing World," or for articles describing nursing experiences, or dealing with any nursing question from an original point of view. The minimum payment for contributions is 5s., but we welcome interesting contributions of a column, or a page, in length. It may be added that notices of entertainments, presentations, and deaths are not paid for, but, of course, we are always glad to receive them. All rejected manuscripts are returned in due course, and all payments for manuscripts used are made as early as possible at the beginning of each quarter.**

### The Royal British Nurses' Association.

ADDRESS BY MRS. HUMPHRY WARD.

ON Thursday afternoon last week the new premises of the Royal British Nurses' Association at 10 Orchard Street were open for inspection, and tea was dispensed in the charming little tea-room on the second floor, where a number of nurses sat and chatted, as well as in the club room, which is close by. Both rooms are papered with a light green and white striped paper, and there are light buff papers on the ceilings. The staircases are a warm red. The chairs and sofas are large and comfortable, and every inducement to sit and rest, read or write, is offered by this cosy suite (if two rooms may be so called). The club room looks out upon St. Thomas's Church and a courtyard with trees, while the tea-room is in the front of the house, overlooking Orchard Street. There is a private entrance to the premises, which are thus quite independent of the saddler's shop on the ground floor. On the walls are appropriate pictures, etchings of the principal hospitals, and photographs of well-known matrons and sisters; and in the tea-room was noticed a case of "Every Requisite for Nurses" from Messrs. Garrould. The rent of the new premises is rather less than that paid for the rooms in Old Cavendish Street, including the cost of hiring the rooms of the Medical Society for meetings, so that the Association incurs no additional liability by the change beyond the cost of removal.

A CHAT WITH MISS ISLIP.

Next door to the tea-room Miss Islip has her office. She is the secretary of the new Auxiliary Society, and very interesting work she finds it. In the interval between tea and the discussion announced for 5.30 there was time for a little chat with her about her new work, as we sat in the office, also papered in light green and white stripes, and furnished partly with some old oak furniture belonging to Miss Islip, who lives on the premises.

"It would never do," she said, "for no one to be here at night, for although we do not advertise night work, it must sometimes happen that a nurse is wanted, and if I am on the spot much valuable time may be saved, more especially when we have the telephone fixed."

"How many nurses have you on the books?" I asked.

"Twenty-two have already been elected on the staff, and I expect about twenty more will be added at the next meeting of the Auxiliary Society. They are all members of the Association."

"Nurses over forty may join, may they not?"

"Yes, that is our object; we have some up to fifty on the books."

This is not the first undertaking of which Miss Islip has had the starting, for she was matron of the Royal Eye Hospital, St. George's Circus, for several years after its rebuilding. She has also had much experience in hospital work at the London and several other hospitals, and was giving a course of massage teaching to the nurses of the Nottingham Hospital when she obtained her present appointment some weeks before Christmas.

MRS. HUMPHRY WARD ON CRIPPLED CHILDREN.

Leaving this office one comes out upon a small landing, from which one flight of stairs leads up to the kitchen, caretaker's room, and the bedroom occupied by Miss Islip; while the other leads down to the two large rooms used as the offices of the Association: they are separated by folding doors. In the inner room Miss Leigh works, while her assistants occupy the other. On Thursday the doors were open, making a room 40 feet long by 18 feet wide, and it was well filled with nurses and friends to hear Mrs. Humphry Ward on "The Education of Crippled Children."

Miss Georgina Scott introduced the speaker, with whom she said, the audience had doubtless often passed many pleasant hours, though they had never before met her personally in this social way.

Mrs. Humphry Ward said that she very much wished to take counsel with those present on the subject of schools for invalid children. She proposed to deal with some modifications arising out of the reading of her paper at the meeting of the National Union of Women Workers in the autumn. The school at the Passmore Edwards Settlement in Tavistock Square was opened in February 1899, and the whole question, of training crippled children had since assumed such importance that the School Board had carried through an enquiry relating to 600 children; a fuller and more exhaustive enquiry was being carried out with the help of a list which by no means exhausted the number of such children living in London. Bristol and Liverpool had also taken the matter up, and at the latter were some appliances which the Tavistock Square authorities greatly envied. These Mrs. Humphry Ward spoke of again later as having been invented by Mr. Harrison, a young surgeon in charge of the children: they were intended to prevent the strain to the eyes by raising the children sufficiently on their couches during lessons.

Mrs. Ward said she looked forward to the time when there would be in London twenty or thirty of such schools. She did not, however, wish to claim that the Passmore Edwards School had been the first in the field; Mrs. Pilcher of Stepney, that invaluable association the Invalid Children's Aid Association, and the Women's Settlement in Southwark had all done something in the same direction; but it was the fact of having a large and beautiful building at Tavistock Square, used only in the evening, that made the committee ask what was the best use for it in the daytime. Two things at once became evident: first, that the classes must be put under the London School Board; and secondly, that they must be more elaborately equipped than those previously in existence. And there must be an ambulance and a nurse. They began by consulting the Invalid Children's Aid Association; next they applied to the hospitals in the neighbourhood, and several doctors, especially Sir Thomas Barlow, gave valuable help. The latter gave the ambulance, which was constructed to carry two lying-down children and eight sitting up, as well as the nurse; the cost was £100. There were now 45 names on the register. The chief diseases from which the children suffered were hip disease, spinal complaints, heart disease, epilepsy and paralysis, rickets, water on the brain, and chorea.

Mrs. Ward next dealt with certain lines of criticism raised by those who thought (1) that such children ought to go to the special schools provided by the London School Board, and (2) that with a little extra care and the provision of special seats they could either go to the ordinary school, or they were too ill to go to school at all. First, no one, she said, knowing both invalid schools and special schools could really support such a theory: it was wrong (and now, since a conference of the London School Board in October, impossible) to mix cripples with the mentally deficient, since they were often rather above than below the normal standard. And she quoted a number of cases in support of this. "God Almighty took away my legs," sobbed one little girl who had been taken from the invalid school to the special school, and put with what she called the silly children, "but He didn't take away my head." With regard to the second point, Miss Lucy Haines (Tower Hamlets Division) had proved that with care, the provision of special seats, and the supervision of the teacher, a certain number of children could go to the ordinary school. But there were others who were only able to work intermittently, and who required medical supervision, they were frequently in hospital; and for such the invalid school was a necessity.

THE NURSE IN THE INVALID SCHOOL.

The nursing was a special point on which she wished to speak. She thought that a nurse who was no longer able to bear the strain of ordinary work might with advantage turn her attention to the invalid school. Her services were demanded from 8 a.m. to about 5.30 p.m. They consisted in fetching the children from their homes in the ambulance; school began at 9 o'clock, but by the Board's permission the registers need not be closed till later, to allow time for all the children to arrive. During lessons the nurse was in attendance in another room, ready to come in if wanted; in the dinner and recreation hour she was responsible, with the help of two or three ladies, and she took the children home, beginning at 3 o'clock in winter and 3.30 in summer. The hardest part consisted in regulating the amusements, as the children had all the spirits of healthy boys and girls without the physical power of giving them vent. It was surprising and very encouraging to know that, besides improving mentally and morally, they had been found to get stronger physically under the kind care of teachers and nurse. The nervous relief of some, when they learnt that they need not be afraid of being roughly treated or overtaxed, was remarkable.

The nurse was free after 5.30, and on Saturdays and Sundays. It was not a sufficiently exciting or interesting work to attract those in full vigour; but she thought a nurse, say after forty years of age, might feel drawn to it: her experience would be very valuable, and she might see possible developments. There were opportunities for personal influence, which might radiate to the parents; and for much womanly sympathy. As to ultimate results, Mrs. Ward said she hoped to see a diminution of the crippled population. When the 2,000 or 3,000 crippled children in London were properly dealt with in twenty or thirty schools much neglect might be prevented. A certain number would never improve, but they must be taught to earn their living; and even if the weary hours of suffering were but made easier, the work would not be thrown away.

AFTER-TRAINING AND THE NURSE'S SALARY.

In the brief discussion which followed two points were raised: that of after-training, by Miss Bowditch, and of the nurse's salary, by Miss Merrick.

As to the first, Mrs. Humphry Ward said the question was receiving a great deal of consideration, and she believed that the County Council would be very willing to co-operate in offering small scholarships. Two or three children would shortly be fit to be placed in schools of design; and there were openings in electrical work, silver chasing, &c., for boys, while needlework at present offered the largest opening for the girls. It was on local committees at the various schools that the responsibility of this must rest. The ambition at Tavistock Square was to make all who were physically fit capable of earning their own living. Local committees must be granted wider powers than those of school managers. In answer to the suggestion as to training the girls as cooks, Mrs. Ward said there were none strong enough in the school.

The nurse's salary was £50, out of which she boarded and lodged herself; Mrs. Ward would, however, be sorry if that was the limit. Under the Act of 1899 the School Board was able to supply "guides and conveyances"; there was probably no notion when the words slipped in that they could be interpreted to mean an ambulance or nurse. The question of the latter at the new schools in contemplation was still undecided, but she hoped that a trained nurse would be insisted upon, and that ultimately the provision of both would rest with the Board, which already bore the expense of the ambulance.

Miss Pritchard having proposed a vote of thanks to Mrs. Humphry Ward, the latter said she would be pleased, on receipt of a post-card addressed to her at the school, for anyone interested to visit the children during dinner and recreation time in order to see something of the work being done.

### The Nurse without a Petticoat.

Dedicated to the R.A.M.C. Orderly, and to his Unpensioned Volunteer Comrade.

There's a toiler all unhonoured, there's a patriot without praise,
There's a hero whom no newspapers applaud,
For the dangers of his mission gain but meagre recognition,
And a distant nameless grave is his reward.

Not for him fierce joy of battle while the dreadful maxims rattle,
Not for him heroic charge nor ringing cheer:
His the tireful dreary bending over stretchers, sights heart-rending,
Noisome smells, and mournful sounds most sad to hear.

Drenched with rain-storms, choked in dust-clouds, soaked in puddles, scorched with heat,
Aching limbs now numbed by frost, now plagued by flies,
Bruised with tent-pegs, rarely sleeping on that couch with vermin creeping,
Toils he, cheerful, uncomplaining, till he dies.

Scanty training he possesses, little use of soap professes,
(There's one basinful of water to a tent)
Hard his hands, unschooled his greeting, but a royal heart is beating
'Neath that khaki-coloured tunic soiled and rent.

Volunteer he, quitting cottage far away across the seas,
Leaving wife and wages at his country's call:
Yet he wins no glowing "mention in dispatches," earns no pension,
Nor the soldier's meed of glory should he fall.

Now the doctor stark is lying, of the Sisters two are dying,
Tents are crowded, comrades down, no time for rest;
Till himself begins to sicken, falls amid the fever-stricken.
All is over. Fold his hands upon his breast.

G. C. G.

### Minor Appointments.

CHILDREN'S INFIRMARY, LIVERPOOL.—Miss Violet Paget has been appointed staff nurse. She was trained at the Infirmary, Birmingham.

EAST LONDON CHILDREN'S HOSPITAL, BOGNOR BRANCH.—Miss Ada Eldridge has been appointed staff nurse. She was trained at Paddington Green Children's Hospital.

CLAYTON HOSPITAL, WAKEFIELD.—Miss Jane Phelps has been appointed night superintendent. She was trained at the Clayton Hospital, and was subsequently sister in the women's wards. She has also been doing private nursing for the Queen Victoria Nursing Institution, Wolverhampton.

PORTSMOUTH WORKHOUSE INFIRMARY.—Miss Minnie Blaker and Mrs. Kate Butler have been appointed charge nurses. Miss Blaker was trained at St. Olave's Workhouse Infirmary and was afterwards on the staff of the Kent Nursing Institution. Miss Butler was trained at the Croydon Workhouse Infirmary.

HERTFORD BRITISH HOSPITAL PARIS.—Miss Adelaide S. Bolton has been appointed charge nurse. She was trained at St. Luke's Hospital, Halifax, and has since been charge nurse at the Memorial Hospital, Jarrow-on-Tyne. Miss S. E. Armstrong, Miss Lucie Howard, and Miss Constance Lunn have been appointed staff nurses. Miss Armstrong was trained at the Royal Infirmary, Derby; Miss Howard at the Fever Hospital, Islington, and the Royal Infirmary, Derby; and Miss Lunn at Hampstead Hospital.

KINGSTON UNION INFIRMARY.—Miss Joyce Williams, Miss Julia Haigh, Miss A. McManis, and Miss J. E. Smith have been appointed ward sisters. Miss Williams was trained at the Croydon Infirmary. She was afterwards assistant-nurse at the Woolwich Fever Hospital, and has since been engaged in private nursing at San Remo. She holds the L.O.S. certificate. Miss Haigh was trained at the Union Infirmary, Walsingham, and holds the L.O.S. certificate. Miss A. McManis was trained at Westminster Hospital, and has since been sister superintendent at the Sudbury Union Infirmary. Miss L. E. Smith was trained at the Union Infirmary, Sunderland, and has since been sister at the Union Infirmary, King's Norton, Birmingham. She holds the L.O.S. certificate.

### Echoes from the Outside World.

AN OPEN LETTER TO A HOSPITAL NURSE.

SORROW has fallen upon all of us: men, women, and children mourn together, for she who has been called away was not only Queen of our land, but also queen of our hearts. The spontaneous tribute of a little child of four was touching in its simple truth. She heard her parents speaking of the likelihood of death, and said tearfully, "Oh, mother, don't say she is going to die! She is such a dear Queen!" And dear indeed she was, and ever will be, to those whom she watched over, and thought over, and prayed over, for so many long years. As far back as last November, when, as is now known, she began to feel her strength failing, it was love for her people which prevented any tidings of ill health from reaching them, lest the prospect of losing their Head should cause them grief before the time. Day after day the Court Circular, which till Friday last she always edited herself, was persistently silent as to any failure of power, any suggestion of illness, though the expression of sympathy for those who were suffering or bereaved came readily enough. As recently as Wednesday of last week she still had the power to perform one of those numerous acts of kindness whose sweetness will always cling to her name. Amongst the choristers at the private chapel at Osborne was an alto ill of a dangerous malady, and the case was brought to the notice of the Queen by Sir Fleetwood Edwards. On Thursday a communication was received by the sufferer to say how distressed Her Majesty was at the news, and enclosing a cheque for £5.

EVERYWHERE arise the same tender memories. Those who were brought into daily contact with Queen Victoria naturally loved her; but there are thousands of others, not only in England, but far away in remote countries, who perhaps never saw her in the flesh, and yet can tell of the kindly action, the womanly thoughtfulness, which so quietly and unostentatiously stole away the hearts of her subjects. In the East End of London, amongst the squalor and poverty which one fancies might easily have deadened feeling, the tears flow as readily, the voices are as hushed, as if the Queen had been a personal friend. Elsewhere the school children feel the sadness in the air, and walk quietly along instead of skipping merrily down the streets; the shopkeepers supply their customers almost in silence, as if voices were hardly to be trusted; and around every fireside there is only one subject to which allusion is made—our irreparable loss. From every quarter of the globe sympathy and sorrow are flashed along the wires, and it is comforting to feel that she whom we loved so much was also beloved both by the subjects of her realm on the other side of the globe and by those to whom she was only the monarch of the greatest power in the world.

PERHAPS the most succinct summary of her character was made by General Miles in New York on Monday. He said that "Queen Victoria was the queenliest Woman and the womanliest Queen in history." And then he went on to speak of her deep love for her soldiers, and theirs for her. Poor fellows! how they will grieve when the mournful intelligence reaches them—those who are still fighting as well as those who are in hospital, or laid by for the time at least, as the result of their services to the land for whom she cherished so deep an affection. It was pleasant to note that when the Queen was sleeping on Monday the Prince and Princess of Wales—now the King and Queen of England—the Kaiser, and other royal personages went to the James Convalescent Home for Invalided Soldiers, close to Osborne, and had a chat with the men in the wards. Had the Queen known, nothing would have gratified her more; as also that prayers for her recovery ascended from the last place to own her sway—the capital of the Transvaal. The war has already created more desolate homes than can be numbered. Alas! that it should also have robbed us prematurely of our Queen. For we all know how that tender heart was moved by the sadness and the distress of the prolonged fighting; and Earl Roberts, when he dined with her on the Monday before her illness, must have deeply regretted that he could not hold out more definite hopes of peace. He was her last visitor, and it must have pained him much that he could only tell her the truth.

ASSUREDLY, love of self occupied a very small part of the affections of our illustrious Queen. For a few years after her great trouble, when her husband was taken from her, she seemed unable to mix in Society, deprived of the stay and help upon which she had learnt to lean, but gradually she came back to the world, and during the last few years she was more amongst us than even in her younger days. Her many appearances in London last spring and summer, just at a time when all the land was sad, was an inspiration, and made her people feel how truly she was one of them. Since then I have never heard man, woman, or child, speak of her except in terms of love and admiration, and every few days, ever since the disastrous South African war commenced, fresh deeds of kindness were done alike to rich and poor. And her life was made up of such acts. On Tuesday I was conversing with an ex-policeman, who, in his younger days, had been a boy about the gardens at Windsor. He and his brother were both employed in the Royal service, and the latter, being a particularly clever whistler, at the suggestion of John Brown, surreptitiously taught one of Her Majesty's favourite parrots to whistle. This he accomplished in the dark, as he found the bird learnt far more quickly when he had nothing to distract his attention, and after he had mastered "God Save the Queen" the bird was persuaded to pipe to Her Majesty. The Queen was very pleased, asked who had been his teacher, and personally praised the lad's patience and the pains he had taken. A few months afterwards she noticed the absence of the boy, and upon enquiry heard that he had died suddenly from pneumonia. Finding that his wages had gone far to support his widowed mother, she not only paid the funeral expenses, but made the woman a present of £50, which was distributed to her weekly until the other children were bigger and able to help. Both the boys attended the Queen's Sunday-school, held, by her desire, in the afternoons for her young servants, and the ex-policeman shows, with much pride, the Bible given by the Queen herself for regular attendance.

IN thinking over the Queen's life I have always been greatly impressed with the lack of privacy and quiet which is one of the penalties of Royalty. All who were brought in contact with her laid stress upon the simplicity of her mode of living and of her habits generally, notwithstanding that she could be very regal when occasion required. To such a character the feeling that she could go nowhere without attracting attention—and attention which it was her duty not to ignore—must often have been a trial. Upon one occasion some years ago in Scotland, she arranged to visit a Scotch nobleman in a purely private way, and proceeded part of the journey by train, the rest of the distance being accomplished by carriage. When she alighted at the tiny wayside station it was nearly two o'clock, and she had evidently been lunching *en route*. Obviously, too, Her Majesty had afterwards indulged in a few minutes' siesta—her start had been an early one—and I could not help being very sorry as she emerged on to the platform, crowded from end to end with countrymen and women, many of whom had walked miles over the hills for a peep of England's Queen, for her eyes were still full of sleep, and yet she had to bow and smile with no regard to her personal feelings.

### Croydon Infirmary Crisis.

By Our Own Correspondent.

Up to the present the Local Government Board have expressed no views on the dispute between the matron of the Croydon Infirmary and the Board of Guardians as to whether Miss Julian was justified or not in refusing to sign three probationers' certificates. It will be recollected that in consequence of this refusal the Board disassociated Miss Julian altogether from the nursing staff, thus confining her to the more domestic part of her duties.

The matter is, however, slowing approaching a termination. In November last the Board authorised the Infirmary Committee to take steps to appoint a superintendent of nurses.

At the last meeting of the Board the Infirmary Committee were authorised to apply to the Local Government Board for consent to the appointment of a superintendent of nurses, and at a meeting of the Board on Tuesday it was stated by the Chairman of the Board that an application for this consent had already been forwarded.

On Tuesday, in accordance with notice, Mr. A. G. Sibun moved that the Board rescind the resolution passed in November last instructing the Infirmary Committee to take steps to appoint a superintendent of nurses. His object was, he said, to get the Board to give up the idea of employing a third officer. They already had a superintendent of nurses and matron of the infirmary, who, it was true, agreed with none of them, or they with her, and he thought the more straightforward way would be to ask Miss Julian to resign. To appoint a third officer would be to throw a fox into the middle of the farmyard. Instead of beating about the bush, as the Board were doing, it would be more straightforward to ask Miss Julian to resign, because if she turned into an angel she could never govern the infirmary satisfactorily after what had transpired, and it was better that she should leave. He believed that the Local Government Board would say that it was wrong to appoint a third officer, and therefore Miss Julian must go.

Mr. F. Wustemann spoke in favour of Miss Julian. He thought she had a right to maintain her position when people came and interfered with her who knew nothing about her duties.

Mr. W. H. J. Owen once more objected to this rash expenditure. The question had not been dealt with in a proper way. Either Miss Julian was competent to do the work of lady superintendent, or she was not. If not competent, she ought to be called upon to resign. If she was competent, but would not do the work—and they knew that she was competent—then she ought to be reported to the Local Government Board. The importation of a superintendent of nurses, when they already had a matron and an assistant matron, would not conduce to the harmony or good working of the infirmary.

Mr. Shirley said if the Board did away with the infirmary being a nursing institution, and ceased to have probationers, it would mean an extra expenditure of £700 or £800 a year for nurses. The present system of probationers was much cheaper. The cost of the superintendent of nurses put at £140, including rations, was only one thirty-second part of a penny on the poor rate; and he asked them to look at the advantage the Board would receive in the training of the probationers. All that they were doing was asking Miss Julian to give up part of her duties, and the Local Government Board had no right to interfere with this.

Mr. Owen said Mr. Shirley in talking about giving up the system of training probationers was taking the Board off the track. No one had raised the question of doing away with the infirmary being a training school.

Mr. Sibun wished the Board to ask Miss Julian to resign, and put another officer in her place, and not to appoint a fresh officer as superintendent of nurses.

There voted for rescinding 8, and against 12.

This is a confirmation of the Board's action applying to the Local Government Board for permission to appoint a superintendent of nurses.

Mr. Sibun intimated he should take further action.

### Everybody's Opinion.

### SO-CALLED TRAINED NURSES.

"M. M. M." writes: In answer to your correspondent "M." I wish to give a case in point. Some time ago I had occasion to send to a nursing institution in one of our large cities for a male nurse for a case of pneumonia. The man they sent had never been inside a lunatic asylum, not to mention a hospital, and could not even take the patient's temperature. Surely such conduct on the part of the proprietor is criminal. The patient, of course, died. This is only one case of many.

### A NOVEL DECORATION.

"Accuracy" writes: The public are much indebted to the writer of the description of "a novel decoration for Christmas, in the shape of a large wire spider's-web, surrounded by ivy, with an artificial spider in the centre." But the statement that "Christmas festivities were resumed at Yeovil District Hospital this season, after a lapse of some years," is a mistake. The patients have always had suitable treats provided for them at Christmas by the generosity of many sympathetic well-wishers of this valuable institution, under the kindly management and co-operation of the matron and nurses. Those interested in the late matron will be glad to hear that she is much appreciated in the new appointment she has held for the last year for her efficiency and courtesy. The committee of her hospital have complimented her on her management, and to mark their satisfaction asked her acceptance of a handsome cheque as a Christmas gift.

### THE DEGRADATION OF NURSING.

"E. E." writes: Under this heading an ex-nurse points out that unqualified folk are teaching in School Board classes and ignorant persons are writing silly booklets. I do not think she can know much on the subject, though it may be so at the two places she refers to. I could mention a booklet edited by a B.A. Oxon., M.R.C.S., L.R.C.P., who also attends the schools to lecture and instruct, and this is not a rare case. Anyone going to the Board School classes will find there is a qualified assistant teacher one week who has to go through a special course, and a registered practitioner takes it each alternate week. If they are the so-called ignorant, of what use are doctors? As there is a staff of eighty medical men who attend these classes, evidently there must be some mistake about the man referred to. It might have been so once—that I cannot say. All I know is that at the present time it is very different.

### NURSING THE SICK AND WOUNDED IN CHINA.

"A King's Nurse" writes: A notice appeared during the month of September in your columns about the nursing of the wounded at Wei-hai-wei, and a letter from a Hong Kong correspondent stated that Miss Gladwell, one of the nurses of the Shanghai Municipal Nursing Home, assisted Miss Batchelor and Miss Barr, who were sent to Wei-hai-wei from the Civil Hospital, Hong Kong. I should feel obliged if you would contradict this statement. Miss Gladwell was at Wei-hai-wei on a holiday when it was decided to open a hospital there for the wounded, and she was asked to help in organising the work and to take charge of the nursing under Drs. Sutton and Reid, R.N., and Major Clark, R.A.M.C., which she did till her holiday expired and she was recalled to Shanghai. Miss Gladwell was trained at King's College Hospital, where she worked for seven years, after which she took up private nursing. Four years ago she was one of the nurses selected to go to Shanghai, where her work is a credit to herself and her training school.

### HOTEL NURSING.

"B. P." writes: It often falls to the lot of a private nurse to be sent to a hotel. A few days ago my present patient was discussing "nurses" and "nursing," and he said that a short time ago he was staying at the Hôtel Métropole and sent for a nurse. She arrived, and almost the first thing she said was: "Where am I to have my meals?" He rejoined, after a little consideration, "With the housekeeper, I should think." Whereupon she immediately left him. Why will nurses be so foolish? If she had only left the momentous question of "meals," and had quietly suggested later on having them in her own room, it would have been infinitely more tactful. It is next to impossible for nurses to go down to table d'hôte. In the first place the proprietor raises objections—and very properly—on the ground that it scares visitors away to see a nurse on the premises. Some people are morbidly inclined, and to know and be constantly reminded that there is sickness about causes dissatisfaction. Secondly, to remove uniform for private clothes takes time, and then to sit out a long dinner would be inconvenient. If people can afford to stay at a hotel and employ a nurse, they can usually pay the little extra for the nurse's meals to be brought to her room. I have nursed in several hotels, both in London and abroad, but never, up to date, have I had any trouble about my meals.

### WARD SISTERS AND PROBATIONERS.

"C. F. Y." writes: I have read Miss Twining's letter with great interest, especially what she says of probationers. The over-work which obliges some girls most reluctantly to give up nursing depends very much on the ward sister. If she is conscientious and judicious, probationers pull through the crucial first month, and gradually become accustomed to the standing, lifting, and other general fatigue. But the ward sister is sometimes an irritable or ill-tempered woman, and one I know seems to have a special dislike to probationers. She forbids any sitting down during the nine hours in ward, and constantly "harries" the probationer, calling her "stupid," "slow," &c. She is unpleasant to the nurses, but not quite to the same degree. Yet her word alone is taken to decide that a probationer is unfit for nursing, and the girl's career as nurse is thereby stopped, as it is not etiquette for another hospital to receive her; though I know one probationer, whom the ward sister pronounced quite useless, who was taken on at another hospital simply because the head knew that sister and mistrusted her fiat. The probationer is now one of the best younger nurses in the hospital. It is perhaps impossible for the matron always to know which women are fit to be in authority; but when the sister's verdict is frequently against probationers, it would be only right for the matron to change the probationer to another ward, and take a second opinion as to her capabilities. I have many friends who are successful nurses, some also who have had to give it up, and they all agree that a "good" or "disagreeable" ward sister makes the utmost difference in commencing nursing.

### PROBATIONERS IN NURSING HOMES.

"A LATE Lady Superintendent" writes: Notwithstanding the frequent warnings given in your pages to those wishing to be trained as nurses, it is to be feared that too many young people are still induced to enter private nursing homes as probationers on the offer of training being given there. In an article on nursing homes in a recent number of the *British Medical Journal*, it is stated that in one home, where the fee charged was £10 10s., the only nurses were two, whose sole knowledge of nursing had been gained in the home itself. A home where the idea of the quiet needed for an operation case was so primitive that the constant noise of footsteps was allowed to sound along uncarpeted passages and stairways, a knocker to be used on the front door, and the peaceful sleep of the patients further disturbed by the cheerful sound of a food lift rattling up and down in the passage close by! How the two so-called nurses gained even a rudimentary knowledge of nursing it is difficult to say, as when a patient came for an operation two "special" nurses were engaged from outside. A short time ago a young girl was induced to go into a similar home, and to sign an agreement to give her services for two years in return for full training. Thinking this meant at least some training in a general hospital, she signed, only to find that she was expected to do the humbler tasks of the sick-room, in addition to much mending, while she picked up her knowledge of the gentle art of nursing by occasionally being left in charge of a patient who probably was paying for the services of a trained nurse. If only the many young people desirous of taking up the profession of nursing could consult some matron skilled in the ways of the world as well as in nursing, they would be saved much disappointment and waste of time, and would be spared from falling into the hands of women who think more of money-making than of spoiling the career of another woman, or of sending out into the world as trained nurses those who are wholly unqualified for the work.

### THE RULES IN PRIVATE NURSING.

"B. P." writes: It seems positively absurd for a nurse to ask public opinion about the times she should give medicine during the night. The doctor in attendance is surely the only person to decide, in the first instance, whether the patient is to have medicine at all through the night, and, secondly, if he should be wakened for it at certain hours. One hard-and-fast rule cannot apply to all cases, as often sleep is much more beneficial than medicine. Then, in reference to the second question, I have been private nursing for some years, and have never yet been asked to sleep in a male patient's room. However, if necessary, and for the patient's benefit, I should raise no objection. But during convalescence a more or less solid diet is usually given, so there can be little or nothing to necessitate a nurse actually sleeping in the man's room. I think with many others that "Watcher" has either written merely for the sake of writing or else that she is not gifted with much common sense.

"FIDELIS" writes: I have worked as trained nurse for six years in this branch, and perhaps my experience may be of use to beginners. It was with great chagrin that I read the subject of "propriety and impropriety" in the treatment of our patients having been raised. I always try to look upon the sick under my charge as a good mother would regard and act towards her suffering sons and daughters, and considering, as much as is practicable, the convenience of their relations, saving expense, &c., as far as possible, letting them see that one does not regard patients solely in the light of cases out of which we are to get as much knowledge, &c., as may be, but that we are truly sorry for their sufferings. So far from having found that nurses have got "a bad name," I believe we are considered very useful members of society. My experience has also been fortunate as to matrons. I never came across one in hospital or a nursing home who more or less did not lay down a good code, and encourage in every way work of a high standard. I think it is a pity that one of our profession calling herself Sister should entertain, let alone publish, the idea of "nurses getting a bad name" and matrons being lax. I find it a good plan in private nursing to keep two ordinary lined copy-books, one for doctor's orders (to be written, if possible, in his presence) and another subdivided so:—

*Day Report.*

| Time. | Food. | Stimulant. | Medicine. | Notes. |
|---|---|---|---|---|
| | | | | |

*Night Report (ditto).*

This saves the doctor from having to ask a lot of questions, and the nurse the minute work of keeping a chart, unless specially ordered to do so. Each entry should be made at once on giving food, stimulants, &c. I am entering on my tenth year in the nursing profession, and I have never heard a word breathed against my profession. I believe in the old saying, "You may as well hang a dog as give him a bad name." "Sister G." would do well to study our national motto, "Honi soit qui mal y pense," and to remember that "to the pure all things are pure."

### Wants and Workers.

NURSE POTTERDON, Burton Villa, Middle Road, Bournemouth, W., would be very grateful for copies of THE HOSPITAL passed on to her as soon as done with.

### TRAVEL NOTE.

CONVENT IN BRUGES (L. A.).—Thank you for your interesting information. I know the convent by repute, but have never been there myself, and it is always most useful to me to receive trustworthy personal evidence concerning foreign places of residence. It seems a most generously-arranged menage.

### A Book and its Story.

### A GREAT AND GOOD WOMAN.

AUGUSTA, EMPRESS OF GERMANY. By CLARA ISCHUDI. Translated by E. M. COPE. (London: Sonnenschein. 1 vol.) Price 7s. 6d.

AUGUSTA, first Empress of Germany and consort of William the First, is comparatively little known to English readers, although a noble and distinguished woman, whose character, developed by trial, was made perfect through suffering. A daughter of Frederick Charles, hereditary Grand Duke of Weimar, and Maria Paulwona of Russia, she was born at Weimar in 1811. Her marriage, by which she was destined to become the wife of one emperor and the mother and grandmother of two succeeding ones, took place in Berlin seventeen years later. Brought up in the exclusively intellectual atmosphere of Weimar, a worshipper of Goethe, whose favourite pupil she was, her early days were passed in the comparative seclusion and limited environment of a small German court. Her mother was a woman of strong individuality, unswerving in rigid performance of the smallest details connected with her high position. To her daughters her devotion was unfailing, and in return perfect obedience to her wishes was exacted. This was willingly given, except on occasions when the zeal of the Princess drove them to the verge of despair, and they ventured to express their weariness, to be met by the reminder "that a princess is not permitted to be tired." Doubtless from the Archduchess Augusta inherited the sterner traits which formed the foundation of a passionate, sensitive nature craving for affection. Unfortunately from circumstances and a natural reserve she failed to find happiness in the one haven where a woman when young expects to find it. Her first meeting with her future consort was "when still but half a child, living in a world of dreams, and therefore surprised at the advent of Prince William, who came to seek her hand. . . . She admired him for the heroic virtues attributed to him, and although he was considerably older than the Princess she was attracted to him by his manliness and self-reliance." Further on we read "that she had looked for happiness in her marriage, but very early days revealed to her that utter dissimilarity of character made this impossible. All her previous illusions were dispelled. She had many attractive qualities. She was clever and refined, and even if she had been less good-looking than she was her youth and dependence upon him ought to have won the heart of the Prince and drawn her closer to him." Opposites attract each other, says Stuart Mill, but it is only harmony that can unite them. In their case, the one force which alone blends discords into harmonious union was, on one side, lacking from the first. Augusta admired and loved her husband; he came to her as the real prince who was to open the doors to a wider, fuller life. But he had no heart to give her. It was given once, and for ever, to the love of his youth, the Princess Radziwill, who for reasons of State he had had to resign. His devotion to her memory is sufficient explanation for much of the sadness of Augusta's married life, and we are told that this devotion was life-long, for "after the death of the Emperor William it was touching to discover the veteran of ninety-one had been used to wear a simple little ring which was on his first finger when he breathed his last, a memento of his early love whose lonely resting place he had been in the habit of visiting for sixty long years. Apart even from so grave a barrier, and one not unknown to Augusta, it was impossible that they could have mingled, for her tastes lay in the world of intellect, of feeling and refinement; his in that of military glory. The picture of Augusta at this date is a moving one:—

"Love and affection had been dealt out to her but sparingly in her childhood, yet she was unfitted for a sunless life: the more lonely and forsaken she felt amid her new surroundings the more frequently she reverted in thought to her old home, where countless interests were always at hand, and where she only needed to ask a question to receive a clever, satisfying reply; but in Berlin and Potsdam she became daily more timid and reserved. Existence here was both unsympathetic and prosaic, without a breath of intellectual life, and she missed daily the proofs of good-will so dear to a woman's heart. The contrast between the little Court of Weimar, with its famous literary traditions, and the military court which was now her home rendered the memories of her early youth the more acute." Three years of married life had passed without any child being born. But on October 13th, 1832, the mother's lonely heart was cheered and the nation overjoyed by the advent of "our Fritz," who became later the Emperor Frederick III. In 1861 William was proclaimed seventh King of Prussia. Augusta was at this time pronounced to be suffering from the incurable malady which "sapping the brightness from her life, was yet powerless to influence the strength of her will. Her health had been broken for years, and no one at that time believed that she would occupy the throne for thirty years, and even survive the Emperor. . . . Time after she submitted to the most painful operations, which brought her to the verge of the grave, and those who loved her wept with sorrow when they learned, after her decease, how many were the scars and seams left by the knife of the surgeon."

In the pursuit of those resources, music and literature, for which she had a natural taste Augusta found some compensation for absence of the loving consideration and understanding which were lacking in those nearest to her. But this could not entirely fill the gap. The craving for affectionate sympathy in her sufferings, which from their very nature isolated her at times, made her turn elsewhere for it. It was at Coblentz, a place dear to her, that her warm heart first found some outlet for its own burden. It was there that she learned to go out of herself, and in entering into the sorrows and sufferings of her subjects to find a solace for her own. In Berlin she had been treated with critical indifference by the people. But the Rhinelanders are more akin to the French, their near neighbours to the West. They have brightness and spontaneous courtesy, without the insincerity which often accompanies it. When Augusta came to Coblentz we are told that she had lost all trust, and had changed from a confiding, joyous girl into a reserved, cautious woman. The warm, frankhearted welcome accorded to her at Coblentz unlocked the frozen channels of emotion, and Coblentz henceforth became the home of her heart. There she gave willing audience to any who claimed her sympathy, and never failed, however suffering, to fulfil when possible any public engagement connected with the hospitals or charitable institutions of the town.

All readers of THE HOSPITAL are probably aware that forty years ago the case of wounded soldiers was very far removed from the present organised system. To fire on an ambulance, to capture army surgeons and their nursing staff, and to ransack hospitals was one of acknowledged usages of war. It was Dr. Palasciano, an Italian, who first raised the important question of the neutrality of the wounded after the Italian war in 1859. From an address delivered by him arose the Geneva Conference in 1863, which resulted in the institution of the "international code," whose badge is the Red Cross. It was with the Red Cross Society that the Empress, then Queen Augusta, specially identified herself. When the Prussian war of 1864 broke out she undertook the direction of the co-operative work by which committees were formed in every German town to collect subscriptions towards the voluntary nursing of the wounded, and to form under her guidance the Union of German Women, who were to work as members of the Red Cross. She visited personally the different military hospitals of several towns and became at home among the wounded at Berlin. In every department she gave invaluable aid both in the erection of temporary hospitals and arrangement of victualling centres. All classes, from princesses to the wives of ministers and generals, working side by side with others of lower social grade, were made one by the common cause which united them. To her also is due the establishment of the first school for nurses in Berlin, where girls of all classes could be instructed. Many daughters of the nobility joined it, and girls who had passed from childhood into a round of Court balls and hollow society routine first learnt from their Queen "that durable happiness can never be founded upon excited nerves, but upon the solid bases of self-sacrifice." The translator has done her part well. The narrative bears no trace of its foreign origin, and flows easily and brightly throughout. The story of the Empress Augusta's life is a valuable addition to the lives of good and great women of the nineteenth century.

### For Reading to the Sick.

### THE GATE BEAUTIFUL.

OUR Saviour's words are, "Watch and pray."
Lord, make us willing to obey—
The wisdom from above impart,
To keep our hand, our tongue, our heart,
In thought, word, deed—that so we may
Pray, while we watch; watch, while we pray.
*J.*

Sweet for each other oft to plead,
And feel our oneness in the Son!
Ah, then we daily meet indeed
In spirit at our Father's Throne!
Our bodies are but parted here,
And fade in this dark land away,
The earthly shadows disappear,
The harvest ripens for that Day.
*Albert Knapp.*

Glory be to God who concedes not one only channel of grace, but many channels; not one only point of access, but many points. Intercessory prayer is truly our Gate Beautiful: outside it sits the halting multitude of our brethren and sisters: we, by God's blessing on our weak walk and endeavour, can enter the Temple through that gate; and not we ourselves alone, but so bringing others with us. Blessed are they who frequenting that Gate, enter by it into the Presence of God; they are making ready for a future day whereon to enter into His presence through a Gate of Pearl.—*Christina Rossetti.*

Have we the least ground for thinking that God accepts coldly and distantly the homage of men's hearts, and acts, and words; that He is not well pleased with it? Is not the joy with which any parent receives the free, habitual service of a child striving to be dutiful, and the submission of one who has been undutiful, the faint image of the joy with which the Father of lights receives him who wishes to dwell at home, as well as the prodigal who has just recollected that he has a home to which he may return?—*F. D. Maurice.*

### FOR ABSENT FRIENDS.

O Lord our God, who art in every place, from whom no space or distance can ever separate us; we know that those who are absent from each other are still present with Thee, and we therefore pray Thee to have in Thy holy keeping those dear ones from whom we are now separated; . . . that whether or not, according as seemeth best to Thy Divine Majesty, we meet together again here on earth, we may surely meet again at the Resurrection of the just, and go in together to that house of many mansions which Thou hast prepared for those that unfeignedly love Thee, through Jesus Christ our Lord. Amen.—*Anon.*

When the hour of death is near us,
Be Thou present, Lord, to cheer us;
In that time of fear and sadness
Tarry not, our help and gladness,
Saviour of the sons of men.

When our latest breath is failing,
Be Thy Spirit all-prevailing;
When the tempter's wiles shall prove us,
Show Thy sacred sign above us,
Hold us, save us, free us then.
—*S. Bernard.*

### Notes and Queries.

**The Editor is always willing to answer in this column, without any fee, all reasonable questions, as soon as possible.**
**But the following rules must be carefully observed:—**
**1. Every communication must be accompanied by the name and address of the writer.**
**2. The question must always bear upon nursing, directly or indirectly.**
**If an answer is required by letter a fee of half-a-crown must be enclosed with the note containing the inquiry.**

*Nurses' Salary.*

(191) My patient died on January 14th. My salary was £30 a year and 2s. washing weekly, and I was usually paid on the 22nd of each month. I was asked to leave on the 19th, and given a £5 note. Should I not have been paid to January 22nd as well as a month's notice?—*E.M.S.*

As your engagement of necessity terminated on the 14th, your salary would be £1 17s. 5d. for three weeks and two days' service, washing 8s., and notice £2 10s. Total £4 15s. 5d.

*Probationers' Agreements.*

(192) 1. Is it legal for probationers to be received for training in any hospital where the Matron is untrained without first being informed of the fact? 2. After agreeing to serve two years as a probationer under certain rules, is it legal for a committee to draw up fresh rules for probationers, and under such rules would they be compelled to stay three years instead of two? 3. If probationers refuse to sign their names to such rules have the committee power to dismiss them? 4. I have unfortunately given eight months' service to an institution, receiving no salary, and because I declined to sign my name to a new "Code of Rules," received my dismissal. Can I claim a testimonial or compensation?—*E.E.E.*

1. Yes. 2. If there was a proper agreement at the outset it would not be legal for the committee at a subsequent period to alter the term of training from two to three years. 3. The committee would have no right to dismiss probationers for merely refusing to sign rules under such circumstances. 4. You cannot claim a testimonial, but if you had a proper agreement you can demand compensation.

*A Workhouse Nurse's Plight.*

(193) After a nursing experience of twelve years, I was appointed nurse to the workhouse infirmary of a country town, where the master proved to be a porter, and the matron entirely untrained. I attended strictly to my own duties, but, after nine months' unpleasantness, the master set it about that "I knew too much for them, and that he would get me out somehow." This he did by carrying untrue tales to the guardians. As I was given no opportunity of clearing myself, I brought an action against the master, and was awarded £125 damages. Since then I have found all workhouses closed against me, and I have now been seven months out of employment, my opponent having written to the masters warning them against me. What redress is there for me? Can the Guardians keep my testimonials from me now?—*Jane F. G.*

This is a hard case, but it is one that may happen to any nurse working under untrained officials. You can obtain no further redress. With regard to obtaining work, you might write to the Secretary of the Workhouse Infirmary Nursing Association, 53 Victoria Street, Westminster, S.W., who would, doubtless, be interested in your case.

*Flannel.*

(194) A sufferer from rheumatism persists, against advice, in wearing flannelette instead of flannel garments. She complains that flannel causes intolerable irritation. Can anything be done for it?—*Trevena.*

Some makes of woollen goods are so exquisitely soft that it is difficult to understand how they can cause irritation. However it is simple enough, if the skin is highly sensitive, to wear an undervest of fine spun silk, and the flannel garment over it.

*Dispensing.*

(195) I am anxious to study dispensing in my spare time. Would you kindly tell me the best books, and say if I can get them secondhand?—*Nurse Don.*

It takes much time and constant practice to learn dispensing satisfactorily. The shilling text-book "Notes on Pharmacy and Dispensing for Nurses" would be as useful to you as any.

*Appointments Abroad.*

(196) Nurse B. would be greatly obliged if the editor would furnish her, at his earliest convenience, with the address of the society in London which finds nurses appointments abroad.

Although most societies send members of their nursing staffs abroad, there is not one for securing appointments for nurses not attached to any association. You might apply to the Hon. Secretary, Colonial Nursing Association, Imperial Institute, S.W. We regret our inability to give our correspondents immediate replies to their questions. The exigencies of space are inexorable. A remittance of 2s. 6d. secures a private reply by post.

*Eccentric.*

(197) Can you kindly tell me where I can find a home for a young woman who is beyond her parents' control? The doctor says she is perfectly sane, but in need of employment. A small weekly sum could be paid for her.—*T. C.*

You say nothing as to the girl's social position, nor of what kind of employment she is capable. In the absence of a private recommendation, select a home from the list of training institutions published in the "Englishwoman's Year-book."

**Standard Books of Reference.**

**"The Nursing Profession: How and Where to Train." 2s. net; post free, 2s. 4d.**
**"The Nurses' Dictionary of Medical Terms." 2s.**
**"Burdett's Series of Nursing Text-Books." 1s. each.**
**"A Handbook for Nurses." (Illustrated.) 5s.**
**"Nursing: Its Theory and Practice." New Edition. 3s. 6d.**
**"Helps in Sickness and to Health." Fifteenth Thousand.**
**"The Physiological Feeding of Infants." 1s.**
**"The Physiological Nursery Chart." 1s.; post free, 1s. 3d.**
**"Hospital Expenditure: The Commissariat." 2s. 6d.**
**All these are published by THE SCIENTIFIC PRESS, Ltd., and may be obtained through any bookseller or direct from the publishers, 28 and 29 Southampton Street, London, W.C.**

